# Joint Clustering and Resource Allocation Optimization in Ultra-Dense Networks with Multiple Drones as Small Cells Using Game Theory †

**DOI:** 10.3390/s23083899

**Published:** 2023-04-11

**Authors:** Tinh T. Bui, Long D. Nguyen, Ha Hoang Kha, Nguyen-Son Vo, Trung Q. Duong

**Affiliations:** 1 Faculty of Electrical and Electronics Engineering, Ho Chi Minh City University of Technology (HCMUT), 268 Ly Thuong Kiet Street, District 10, Ho Chi Minh City 700000, Vietnam; 2 Vietnam National University Ho Chi Minh City, Linh Trung Ward, Thu Duc District, Ho Chi Minh City 700000, Vietnam; 3 Institute of Research and Development, Duy Tan University, Da Nang 550000, Vietnam; 4 School of Engineering and Technology, Duy Tan University, Da Nang 550000, Vietnam; 5 School of Electronics, Electrical Engineering and Computer Science, Queen’s University Belfast, Belfast BT3 9DT, UK

**Keywords:** game theory, ultra-dense network, energy efficiency, unmanned aerial vehicle (UAV), massive multiple-input multiple-output (mMIMO)

## Abstract

In this study, we consider the combination of clustering and resource allocation based on game theory in ultra-dense networks that consist of multiple macrocells using massive multiple-input multiple-output and a vast number of randomly distributed drones serving as small-cell base stations. In particular, to mitigate the intercell interference, we propose a coalition game for clustering small cells, with the utility function being the ratio of signal to interference. Then, the optimization problem of resource allocation is divided into two subproblems: subchannel allocation and power allocation. We use the Hungarian method, which is efficient for solving binary optimization problems, to assign the subchannels to users in each cluster of small cells. Additionally, a centralized algorithm with low computational complexity and a distributed algorithm based on the Stackelberg game are provided to maximize the network energy efficiency (EE). The numerical results demonstrate that the game-based method outperforms the centralized method in terms of execution time in small cells and is better than traditional clustering in terms of EE.

## 1. Introduction

The global mobile data traffic is witnessing an exponential increase, and the number of global mobile connected devices is forecast to increase rapidly from 8.8 billion in 2018 to 13.1 billion in 2023 [1]. The deployment of ultra-dense networks (UDNs) as a type of cellular network is an extremely promising technology for supporting the huge number of connections, especially in the applications of massive Internet of Things (IoT). In comparison with present network technologies, UDNs help to considerably improve the network capacity, spectral efficiency, as well as coverage with a high quality-of-service (QoS) by reducing the distance between the base stations (BSs) and user equipment (UE) [2,3]. However, the highly dense deployment of small cells in UDNs causes high interference, high power consumption, and high computational complexity, which must be addressed to satisfy the stringent constraints of the beyond fifth-generation networks (B5G) [4].

In UDNs, small cells are smaller than ever, and therefore the distance of neighboring small cells decreases. As a result, the intercell interference is extremely high. In addition, the interference is especially hard to control becaused small cells are randomly distributed. There are several studies on interference mitigation in UDNs. In [5], to cope with co-channel interference, the authors used a conflict graph based on machine learning to form a clustering problem and subchannel allocation (SCA) problem for throughput maximization. Two other user-centric clustering methods, using k-means algorithms and jamming strategies, were proposed in [6,7]. These papers show that the clustering-based approach is an efficient tool for mitigating interference in UDNs. However, the clustering methods in [5,6,7] did not consider the signal to interference plus noise ratio (SINR), which directly influences the data rate. This can lead to a decline in the throughput, which is an important advantage of UDNs compared with conventional networks. In [8], an adaptive clustering method was proposed to maximize the spectral efficiency and the network throughput using two stages: offline and online. Additionally, sum-rate maximization was taken into account as the main objective in a user-centric clustering method, a modified Louvain method, proposed in [9].

Owing to maneuverability, using unmanned aerial vehicles (UAVs) in communications is becoming increasingly popular because the networks can be quickly built and have flexibility. Compared with small terrestrial base stations, drones can easily move to form many different structures of networks to serve users more efficiently. Additionally, the channels between drones and users are usually line-of-sight channels because drones fly at high altitudes where obstacles to the users are less likely. It is also convenient for network operators because small cells can be easily added or removed to increase performance or save cost depending on the number of connections as well as the requirements of users. Thus, in communications, using UAVs as small flying base stations is a promising solution to serve hotspots where there is a massive number of users such as at sports events, concerts, exhibitions, and fairs, or provide mission-critical services, e.g., disaster recovery, traffic congestion, etc. [10,11]. UAVs, as flying base stations, can be widely used in many scenarios such as cognitive radio networks [11], ultra-reliable low-latency communications [12], communications using reconfigurable intelligent surface [13], and caching [14]. Despite the advantages of UAVs compared with terrestrial base stations, they still face many challenges in the attempts at employing UAVs in wireless networks. For example, in terms of deployment, the authors of [15] proposed a deployment method for multiple UAVs with the aim of maximizing both the coverage area and the coverage lifetime, whereas a Q-learning method with the reward being the sum of user data rates was used in [16]. Regarding area throughput and energy management, mixed-integer programming problems were formulated and efficiently solved for establishing the optimal location and action scheduling under the constraints of battery and powered energy in [17,18]. Additionally, the authors of [19] considered improving the spectrum efficiency by optimizing bandwidth allocation, power allocation, and trajectory for one UAV. On the other hand, due to the fast movements and jitters of UAVs, there are quick changes in the channels between the UAVs and ground users [20]. Therefore, real-time computing is crucial in UAV-assisted networks, especially in UDNs with many small cells [11,21].

In UDNs, the complexity of optimal resource allocation problems is very high because massive numbers of small cells and small UEs (SUEs) are randomly and densely deployed in large-scale areas. Game theory (GT), which is a distributed mathematical framework, is efficiently used for decoupling an extremely complex problem into multiple subproblems with lower complexity. Thus, there are several studies that investigated the applications of GT in UDNs. In [22], two noncooperative games (NCGs) were designed for solving the power allocation and user association with constraints on the quality of service (QoS). The proposed combination of backhaul game and access game significantly improved the total rate compared with the hierarchical game. Additionally, in [23], the authors considered small cell networks with nonorthogonal multiple access (NOMA) with the aim of network throughput maximization. The results showed the effectiveness of NOMA and the locally cooperative game in interference mitigation in UDNs. In spite of some improvements in [22,23], the main objective was to maximize the throughput; in UDNs, the energy issue is more critical due to the high power consumption of a massive number of network elements. Energy efficiency (EE), which is defined as the ratio of total data rate to total power consumption, is a useful metric for modeling the trade-off between throughput and energy. However, EE maximization problems are nonconvex optimization problems and much harder than throughput maximization problems or power minimization problems. In [24,25],  two optimization problems (i.e., SCA and power allocation (PA)) were considered with PA problems modeled by the Stackelberg game (SG) in order to maximize the EE. Although there were some considerable improvements in terms of EE, there was a limitation in the applicability of the used model of UDNs, which had only one macro base station (MBS) and one BS in a network. In the previous works, either cooperation or noncooperation between network elements was considered, but these elements have the characteristics of both collaboration and competition,. In detail, small-cell base stations (SBSs) prefer to be in joint clusters for cooperatively transmitting signals to UEs. This leads to a decrease in intracluster interference. On the other hand, the SBSs in different clusters tend to conflict regarding power usage. This means that increasing the power of an SBS in a cluster also increases the interference for SUEs in adjacent clusters. Therefore, there is a lack of a game-based paradigm that has the characteristics of both cooperation and competition of network elements in order to concurrently mitigate interference, maximize the network EE in drone-aided UDNs with multiple MBSs, and handle a massive number of randomly distributed drones.

Motivated by the aforementioned discussion, this paper proposes a mathematical model to jointly design the clustering and resource allocation for maximizing the total network EE in UDNs consisting of multiple MBSs and many small-cell drones (SCDs). Cells served by MBSs are macrocells, and cells served by drones are small cells. We propose a coalition game for clustering SCDs to restrict the intercell interference. The SCDs in each cluster cooperatively transmit the signals to UEs. The SCA methods for two tiers are provided to choose the subchannel with the highest channel gain for each UE. To reduce the computational complexity, centralized and distributed PA methods are proposed in terms of maximizing the EE. The main contributions of our paper are summarized as follows:

- Adjacent SCDs tend to join together and cooperatively serve UEs because they highly interfere with each other. Thus, we form this cooperation with a coalition game, with the utility function being the signal-to-interference ratio (SIR).
- To fully eliminate the intracluster interference, we model the SCA for small cells as a binary optimization problem. Then, the Hungarian method is proposed to obtain the solution. Meanwhile, the SCA for macrocells chooses the subchannel with the highest channel gain without any constraint.
- To reduce the complexity of nonconvex PA optimization problems, we propose an iterative centralized PA algorithm to obtain at least a locally optimal solution in terms of maximizing the EE. Inequalities are used for relaxing the complex objective functions of EE maximization into convex functions that are easy to solve with programming tools.
- The distributed PA algorithm based on SG consists of two subcooperative games with MBSs as leaders and SCDs as followers. This algorithm decouples the very complex PA optimization problems into multiple low-complextiy and convex ones that are extremely useful for solving optimization problems with a huge number of variables.

Compared with the conference version [26], a macrocell layer consisting of multiple MBSs is added to the networks. As such, the interference environment is more complex due to intercell interference. Additionally, the objective function is to minimize the network EE, which is more practical to use energy more efficiently compared with the objective of maximizing the sum-rate in UDNs but is more complex to solve because of the fractional-type function. Moreover, we propose a centralized approach to solve PA as a benchmark and the distributed one using the Stackelberg game, and  multiple simulations with different aspects such as EE, sum rate, and execution time were performed to prove the efficiency of the proposed methods. The rest of this paper is organized as follows: Section 2 presents the system model and optimization problems used in this paper. A small-cell clustering method based on the coalition game is described in Section 3. Then, the SCA for both macrocells and small cells as well as the beamforming method for MBSs are discussed in Section 4. In addition, Section 5 describes both centralized and distributed optimization methods for solving PA problems. Numerical simulations proving the efficiency of our proposed methods are outlined in Section 6. Section 7 provides the conclusions of this study.

## 2. System Model and Problem Formulation

### 2.1. System Model

**Figure 1:**
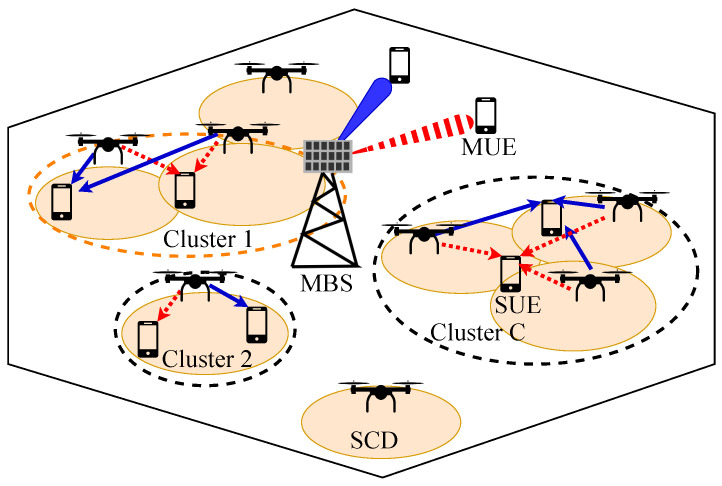
A typical hexagon macrocell in UDNs. Each small cell has an SCD as a flying base station with bounded circle coverage. Dotted red lines and beams denote the signal from BSs to UEs in a subchannel, while the others denote the signal in another one.

In this paper, we consider the downlink transmission in a two-tier UDN that consists of multiple macrocells and a massive number of UAVs serving as small-cell base stations with highly dense deployments. MBSs and SCDs share the same *N* orthogonal subchannels with bandwidth BW per subchannel to serve their UEs. The advantage of using multiple orthogonal subchannels is that multiple UEs can be simultaneously served in these subchannels without interference. One typical macrocell with two subchannel is illustrated in Figure 1. In the macrocell tier, each *F* MBS is equipped with *T* array antennas to transmit the signal to *M* MUEs by using massive multiple-input multiple-output (mMIMO) techniques. In the small-cell tier, to serve *U* UEs, many *B* SCDs equipped with one omni-directional antenna each are randomly deployed according to the independent homogeneous Poisson point processes (PPP) Φ with density λ in the coverage of MBSs (B>U) [24]. When UEs are located in the coverage of one or some SCDs, they are served by SCDs, i.e., SUEs. On the other hand, if the UEs are not in the coverage of any SCD, then the power of SCDs is not high enough to serve these UEs or causes extremely high interference with surrounding SCDs, especially in UDNs. Therefore, they are served by MBSs, i.e., MUEs. We used the main mathematical notation, which is shown in Table 1, to design tbe optimization problems. At each MBS, because of the mMIMO, the number of antennas of MBS *f* is much greater than the number of its authorized MUEs ($T\gg M_{f}$). Each signal $s_{m,f}^{\left(n\right)}$ is multiplied by a beamforming vector $w_{m,f}\in C^{T\times 1}$, and then the aggregation of processed signals of MUEs that are served by MBS *f* is transmitted.

The aggregation $x_{f}^{\left(n\right)}$ at MBS *f* in subchannel *n* is the sum of $M_{f}$ processed signals:

(1) $$x_{f}^{\left(n\right)}=\sum_{m\in M_{f}}a_{m,f}^{\left(n\right)}w_{m,f}s_{m,f}^{\left(n\right)},$$

where the binary indicator of SCA is defined as

$$a_{m,f}^{\left(n\right)}=\left\{\begin{array}{cc} 1, & \mathrm{if}\;\mathrm{MUE}\;m\;\mathrm{is}\;\mathrm{assigned}\;\mathrm{MBS}\;f\;\mathrm{in}\;\mathrm{subchannel}\;n,\\ 0, & \mathrm{otherwise}. \end{array}\right.$$

The received signal $y_{m,f}^{\left(n\right)}$ at MUE *m* served by MBS *f* in subchannel *n* is expressed by

(2) $$\begin{array}{c} y_{m,f}^{\left(n\right)}=\underset{\mathrm{desired}\;\mathrm{signal}}{\underbrace{h_{m,f}^{\left(n\right)}w_{m,f}s_{m,f}^{\left(n\right)}}}+\underset{\mathrm{cross}-\mathrm{talk}\;\mathrm{interference}}{\underbrace{\sum_{m^{\prime }\in M_{f}\setminus m}a_{m^{\prime },f}^{\left(n\right)}h_{m,f}^{\left(n\right)}w_{m^{\prime },f}s_{m^{\prime },f}^{\left(n\right)}}}+\underset{\mathrm{interference}\;\mathrm{from}\;\mathrm{other}\;\mathrm{MBSs}}{\underbrace{\sum_{f^{\prime }\in F\setminus f}h_{m,f^{\prime }}^{\left(n\right)}x_{f^{\prime }}^{\left(n\right)}}}\\ +\underset{\mathrm{interference}\;\mathrm{from}\;\mathrm{clusters}}{\underbrace{\sum_{C_{i}\in C}\sum_{b\in C_{i}}\sum_{u\in U_{C_{i}}}a_{u,C_{i}}^{\left(n\right)}h_{m,b}^{\left(n\right)}s_{u,b}^{\left(n\right)}}}+n_{m}, \end{array}$$

where the aggregation of signals at MBS $f^{\prime }$ is $x_{f^{\prime }}^{\left(n\right)}=\sum_{m^{\prime }\in M_{f^{\prime }}}a_{m^{\prime },f^{\prime }}^{\left(n\right)}w_{m^{\prime },f^{\prime }}s_{m^{\prime },f^{\prime }}^{\left(n\right)}$, and $n_{m}∼\mathrm{CN}\left(0,\sigma^{2}\right)$ is additive white Gaussian noise (AWGN) at MUE *m*.

The received signal $y_{u,C_{i}}^{\left(n\right)}$ of SUE *u* served by cluster $C_{i}$ in subchannel *n* is given by

(3) $$\begin{array}{c} y_{u,C_{i}}^{\left(n\right)}=\underset{\mathrm{desired}\;\mathrm{signal}}{\underbrace{\sum_{b\in C_{i}}h_{u,b}^{\left(n\right)}s_{u,b}^{\left(n\right)}}}+\underset{\mathrm{interference}\;\mathrm{from}\;\mathrm{MBSs}}{\underbrace{\sum_{f\in F}h_{u,f}^{\left(n\right)}x_{f}^{\left(n\right)}}}+\underset{\mathrm{interference}\;\mathrm{from}\;\mathrm{other}\;\mathrm{clusters}}{\underbrace{\sum_{C_{j}\in C\setminus C_{i}}\sum_{b^{\prime }\in C_{j}}\sum_{u^{\prime }\in U_{C_{j}}}a_{u^{\prime },C_{j}}^{\left(n\right)}h_{u,b^{\prime }}^{\left(n\right)}s_{u^{\prime },b^{\prime }}^{\left(n\right)}}}+n_{u}, \end{array}$$

where $n_{u}∼\mathrm{CN}\left(0,\sigma^{2}\right)$ is the AWGN of SUE *u*, $h_{u,b}^{\left(n\right)}=g_{u,b}^{\left(n\right)}\sqrt{\beta_{u,b}^{\left(n\right)}}$ is the channel response, $g_{u,b}^{\left(n\right)}$ is a complex Gaussian random variable with zero mean and unit variance that represents the small-scale fading of the channel from SCD *b* to SUE *u*, and $\beta_{u,b}^{\left(n\right)}$ is the large-scale coefficient of this channel. Therefore, the SINRs at SUE *u* and MUE *m* are, respectively, written as

(4) $$\gamma_{u}=\frac{\sum_{b\in C_{i}}|h_{u,b}^{\left(n\right)}{|}^{2}p_{u,b}^{\left(n\right)}}{I_{u,F}^{\left(n\right)}+I_{u,C\setminus C_{i}}^{\left(n\right)}+\sigma_{u}^{2}},$$

(5) $$\gamma_{m}=\frac{|h_{m,f}^{\left(n\right)}w_{m,f}{|}^{2}p_{m,f}^{\left(n\right)}}{I_{\mathrm{cross}-\mathrm{talk}}^{\left(n\right)}+I_{m,F\setminus f}^{\left(n\right)}+I_{m,C}^{\left(n\right)}+\sigma_{m}^{2}}.$$

### 2.2. Problem Formulation

**Figure 2:**
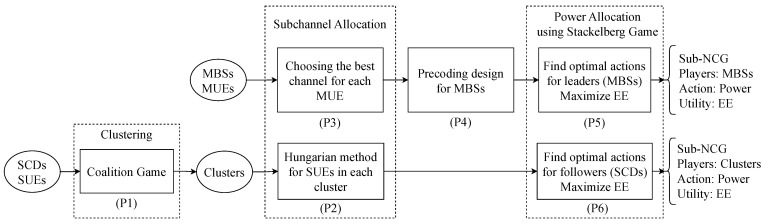
Block processing operations of proposed optimization solutions.

As the deployment of small cells in UDNs becomes extremely dense, the problem of effectively using resources becomes more important than ever. In this section, we formulate the optimization problems for the resource allocation of both spectrum and power according to the processing operations in Figure 2. The overall architectures of the proposed optimization solutions are shown in Figure 2 with the names of the 6 problems in blocks. The detailed description and algorithms are presented in Section 3, Section 4 and Section 5. The objective functions at different stages are different to obtain an optimal solution for multiple proposed objectives, such as interference mitigation in clustering, a guarantee of high-gain channels in subchannel allocation, and efficient energy usage in power allocation.

#### 2.2.1. Subchannel Allocation (SCA)

Due to the high efficiency of spectral usage, co-channel assignment is usually used in practical systems [27]. However, this causes high interference between the two tiers in UDNs and is hard to control, especially with the random deployment of small cells. Thus, the optimization problems with the objective to choose the highest channel gain guarantee that the BSs serve their UEs in good subchannels.

**SCA for macrocells:** We consider MBS *f* serving $M_{f}$ MUEs. By using mMIMO technology at each MBS, the number of MUEs in each subchannel causes a minor interference effect. The target is to find a subchannel with the highest gain for each MUE. The optimization problem can be expressed as

(6a) $$\begin{array}{cc} \max_{a_{m,f}^{\left(n\right)}} & \;\sum_{m=1}^{M_{f}}\sum_{n=1}^{N}{\mathrm{VM}}_{m,n}a_{m,f}^{\left(n\right)} \end{array}$$

(6b) $$\begin{array}{cc} s.t. & \;\sum_{n=1}^{N}a_{m,f}^{\left(n\right)}=1,m=1,\ldots ,M_{f}, \end{array}$$

(6c) $$\begin{array}{cc} \; & \;a_{m,f}^{\left(n\right)}\in \left\{0,1\right\},n=1,\ldots ,N,\forall m\in M_{f}, \end{array}$$

where ${\mathrm{VM}}_{m,n}=\mathrm{trace}\left\{∥h_{m,f}^{\left(n\right)}∥\right\}$. (6b) indicates that each MUE $m\in M_{f}$ authorizes only one subchannel. (6c) represents the constraint of the binary values of the SCA. If $a_{m,f}^{\left(n\right)}=1$, then MUE *m* is served by MBS *f* through subchannel *n*, and vice versa if $a_{m,f}^{\left(n\right)}=0$. Optimization problem (6) is denoted by (P3) in Figure 2. Problem (6) is integer linear programming because (6a) and (6b) are affine functions, and (6c) is the constraint of two discrete values of variables.

**SCA for small cells:** In Figure 2, after clustering the small cells, the SCDs in each cluster cooperatively transmit the same signal to their SUEs. Therefore, the power of the desired signal at each SUE is improved compared with that using only one serving SCD. Considering cluster $C_{i}$, to find the appropriate subchannels for the SUEs in set $U_{C_{i}}$, an optimization problem is formulated as

(7a) $$\begin{array}{cc} \max_{a_{u,C_{i}}^{\left(n\right)}} & \;\sum_{u=1}^{U_{C_{i}}}\sum_{n=1}^{N}{\mathrm{VC}}_{u,n}a_{u,C_{i}}^{\left(n\right)} \end{array}$$

(7b) $$\begin{array}{cc} s.t. & \;\sum_{u=1}^{U_{C_{i}}}a_{u,C_{i}}^{\left(n\right)}=1,n=1,\ldots ,N, \end{array}$$

(7c) $$\begin{array}{cc} \; & \;\sum_{n=1}^{N}a_{u,C_{i}}^{\left(n\right)}=1,\forall u\in U_{C_{i}}, \end{array}$$

(7d) $$\begin{array}{cc} \; & \;a_{u,C_{i}}^{\left(n\right)}\in \left\{0,1\right\},n=1,\ldots ,N,\forall u\in U_{C_{i}}, \end{array}$$

where ${\mathrm{VC}}_{u,n}=\sum_{b\in C_{i}}|h_{u,b}^{\left(n\right)}{|}^{2}$ is the sum of channel gains from SCDs in cluster $C_{i}$ to SUE *u*. (7b), (7c) indicates that each subchannel *n* is authorized by at most one SUE $u\in U_{C_{i}}$ and vice versa. (7d) is the constraint of the binary values of SCA. If $a_{u,C_{i}}^{\left(n\right)}=1$, then SUE *u* is served by the SCDs in cluster $C_{i}$ through subchannel *n*, and vice versa if $a_{u,C_{i}}^{\left(n\right)}=0$. Optimization problem (7) is denoted by (P2) in Figure 2. Problem (7) is integer linear programming because (7a)–(7c) are affine functions, and (7d) is the constraint of two discrete values of variables. The solving methods for (P2) and (P3) are proposed in Section 4.1.

#### 2.2.2. Power Allocation (PA)

In UDNs, the distance between the SCDs and SUEs as well as the distance between small cells are very small: from a few dozen to a few hundred meters. Therefore, increasing the power at an SCD to improve the signal to its UEs causes extremely high interference to other small cells compared with that of conventional networks. In addition, different tiers have different objectives that need to be optimized. For that reason, after SCA, we formulate two optimization problems for PA in macrocells and small cells (Figure 2).

**PA for MBSs** (EE maximization): Each MBS, equipped with massive antennas, is connected directly to a core network. MBSs, using high power with wide coverage areas, guarantee the serving of all UEs that are not connected to any small cell. Therefore, an important problem for MBSs is maximizing the data rate of the MUEs to satisfy the standards of the new generations of future networks (B5G) while efficiently using energy. EE, which is the ratio of data rate to power consumption, is widely used for evaluating the efficiency of energy usage. The optimization problem of PA for MBSs is as follows

(8a) $$\begin{array}{cc} \max_{p_{m,f}^{\left(n\right)}} & \;\frac{\sum_{m=1}^{M}BW\log_{2}\left(1+\gamma_{m}\right)}{\sum_{f\in F}P_{f}^{\mathrm{total}}} \end{array}$$

(8b) $$\begin{array}{cc} s.t. & \;\sum_{n=1}^{N}\sum_{m\in M_{f}}a_{m,f}^{\left(n\right)}p_{m,f}^{\left(n\right)}\leq P_{\mathrm{MBS}}^{\max},\forall f\in F, \end{array}$$

(8c) $$\begin{array}{cc} \; & p_{m,f}^{\left(n\right)}\geq 0,\forall f\in F,\forall m\in M,n=1,\ldots ,N, \end{array}$$

(8d) $$\begin{array}{cc} \; & BW\log_{2}\left(1+\gamma_{m}\right)\geq Tpm_{\min},\forall m\in M, \end{array}$$

where the variable $p_{m,f}^{\left(n\right)}$ represents the transmit power of MBS *f* to MUE *m* in subchannel *n*. $P_{f}^{\mathrm{total}}=P_{f}^{\mathrm{circuit}}+\sum_{n=1}^{N}\sum_{m\in M_{f}}a_{m,f}^{\left(n\right)}p_{m,f}^{\left(n\right)}$ is the sum of power fo ther hardware circuits and power for transmitting signal in all subchannels of MBS *f*, $P_{MBS}^{\max}$ is the maximum power of each MBS, and $Tpm_{\min}$ is the minimum throughput of each MUE. (8b) and (8c) are the constraints of the transmit power of MBSs, which range from 0 to $P_{MBS}^{\max}$. (8d) guarantees the QoS of all UEs.

**PA for SCDs** (EE maximization): Due to the short distance between the SCDs and SUEs, the channel links experience the line of sight (LoS). However, with a large number of small cells, the power consumption rises considerably. Thus, the target of SCDs is effectively using limited energy. The optimization problem for maximizing the EE of small cells is formulated as

(9a) $$\begin{array}{cc} \max_{p_{u,b}^{\left(n\right)}} & \;\frac{\sum_{u=1}^{U}BW\log_{2}\left(1+\gamma_{u}\right)}{\sum_{C_{i}\in C}\sum_{b\in C_{i}}P_{b}^{\mathrm{total}}} \end{array}$$

(9b) $$\begin{array}{cc} s.t. & \;\sum_{n=1}^{N}\sum_{u\in U_{C_{i}}}a_{u,C_{i}}^{\left(n\right)}p_{u,b}^{\left(n\right)}\leq P_{\mathrm{SCD}}^{\max},\forall b\in B, \end{array}$$

(9c) $$\begin{array}{cc} \; & \;p_{u,b}^{\left(n\right)}\geq 0,\forall b\in B,\forall u\in U,n=1,\ldots ,N, \end{array}$$

(9d) $$\begin{array}{cc} \; & \;BW\log_{2}\left(1+\gamma_{u}\right)\geq Tps_{\min},\forall u\in U, \end{array}$$

where $p_{u,b}^{\left(n\right)}$ denotes the transmit power of SCD *b* to SUE *u* in subchannel *n*. $P_{b}^{\mathrm{total}}=P_{b}^{\mathrm{circuit}}+\sum_{n=1}^{N}\sum_{u\in U_{C_{i}}}a_{u,C_{i}}^{\left(n\right)}p_{u,b}^{\left(n\right)}$ is the sum of power for hardware circuits and power for transmitting signal in all subchannels of SCD $b\in C_{i}$, and $P_{SCD}^{\max}$ is the maximum transmit power of each SCD, $Tps_{\min}$ is the minimum throughput of each MUE. (9b) and (9c) represent the range of transmit power. To guarantee the QoS, (9d) is used for all SUEs.

## 3. SCDs Clustering by Coalition Game (P1)

In this section, we introduce a coalition game for clustering small cells. In UDNs, an effective clustering method is essential because of three main reasons, as follows:

- Mitigating interference: Intercell interference is a big challenge in UDNs due to the ultra-dense density of small cells, especially for SUEs in the overlapping areas of small cells. Formulating clusters that combine small cells seriously interfering with each other is an appropriate solution. The SCDs in the same cluster cooperatively transmit the signal to UEs so as there is no intracluster interference.
- Enhancing the quality of the signal: In UDNs, the SUEs in the overlapping areas of two or more small cells are usually far from their primal SCDs, while they receive high interference from the others. In many cases, using some of these SCDs along with primary SCDs to serve UEs helps improve the power of the desired signal. Furthermore, multiple SCDs transmitting the same signal to a UE also improves the diversity gain.
- Decreasing handover processes: The smaller the size of small cells, the larger the number of handover processes. Moreover, when UEs move at high speed, the central controller has to compute the complex handover processes with the massive number of UEs in a short time. Therefore, if small cells join together to form temporarily bigger cells, the number of handover processes significantly declines.

Therefore, adjacent small cells have an incentive to join clusters to obtain a higher value for the objective instead of independent transmission. The coalition game. as a type of cooperative game where players tend to cooperate to achieve higher total utility, is appropriate to model this situation. The standard form of a coalition game is $CG=\langle \mathrm{PL},v\rangle$, where $\mathrm{PL}=B_{\mathrm{active}}$ is the set of players (i.e., SCDs, which serve at least one SUEs), and $v\left(C\right)\subseteq R^{C}$ is the set of nontransferable utility functions of clusters [28]. The utility function of cluster $C_{i}$ is defined as

(10) $$v\left(C_{i}\right)=\sum_{u\in U_{C_{i}}}SIR_{u},$$

where $SIR_{u}$ is the SIR of the links from cluster $C_{i}$ to SUE *u* and is expressed as

(11) $$SIR_{u}=\frac{\sum_{b^{\prime }\in C_{i}}{\left|{\bar{h}}_{u,b^{\prime }}\right|}^{2}}{\sum_{C_{j}\in C\setminus C_{j}}\sum_{b\in C_{j}}|{\bar{h}}_{u,b}{|}^{2}},$$

where ${\bar{h}}_{u,b}=\frac{1}{N}\sum_{n=1}^{N}h_{u,b}^{\left(n\right)}$ is the average of channel gain from SCD *b* to SUE *u*. The solution of this coalition game is the set of coalitions of players or clusters of SCDs in UDNs [28]. In these clusters, there is no SCD that has an incentive to change clusters for achieving higher utility. This is the state where all clusters have reached equilibrium. The two operations that change the SCDs between clusters are split-merge and swap [29]. If SCD $b\in C_{j}$ leaves $C_{j}$ to join $C_{i}$, then the operation is denoted as $\left\{C_{i},C_{j}\right\}\to \left\{C_{i}\cup \left\{b\right\},C_{j}\setminus \left\{b\right\}\right\}.$ In addition, if SCD $b\in C_{j}$ swaps with $b^{\prime }\in C_{i}$, then the operation is described as $\left\{C_{i},C_{j}\right\}\to \left\{C_{i}\cup \left\{b\right\}\setminus \left\{b^{\prime }\right\},C_{j}\cup \left\{b^{\prime }\right\}\setminus \left\{b\right\}\right\}.$ SCD *b* tends to leave $C_{j}$ to join $C_{i}$ or prefer $C_{i}$ to $C_{j}$ if $v\left(C_{i}\cup \left\{b\right\}\right)+v\left(C_{j}\setminus \left\{b\right\}\right)>v\left(C_{i}\right)+v\left(C_{j}\right).$

To obtain the solution to this coalition game, we use Algorithm 1. In this algorithm, we use the variable $d_{\max}$ for determining the SUEs that are less than $d_{\max}$ m away from SCD *b*. Then, the set of neighbor clusters $C_{\mathrm{near}}$ that consists of these SUEs is determined. Therefore, different from [30], we only consider some close clusters with SCD *b* because the interference from *b* has a minor impact on far SUEs at very high frequency. In addition, variables *g* and $g_{n}$ are used for choosing the best option for SCD *b* to move to a new cluster. If the number of SCDs in each cluster increases, then the number of pairs of SCD and SUE with a distance larger than $d_{\max}$ increases. Thus, we use $N_{\max}$ for limiting the number of SCDs in each cluster.
**Algorithm 1** Clustering SCDs using the coalition game.1:**Input:** Channel matrix H, the set of players $\mathrm{PL}=B_{\mathrm{active}}$, locations of SCDs and SUEs, the maximum number of SCDs in a cluster $N_{\max}$, the number of subchannels *N*, $d_{\max}$2:Randomly initialize a partition $C^{\left(0\right)}$, l=03:**while** $C^{\left(l\right)}\neq C^{\left(old\right)}$4:   $C^{\left(old\right)}\leftarrow C^{\left(l\right)}$5:   **for** $b\in B_{\mathrm{active}}$6:      Assume $b\in C_{j}$, $C_{\mathrm{near}}$ is the set of clusters that have some SUEs covered with radius $d_{\max}$ and center *b*,  gap g=07:      **for** $C_{i}\in C_{\mathrm{near}}$8:         **if** $|C_{i}|=N_{\max}$ and $U_{C_{i}}+U_{b}-U_{b^{\prime }}\leq N$ and $U_{C_{j}}+U_{b^{\prime }}-U_{b}\leq N$9:            $C^{\left(tmp\right)}\leftarrow$ swap SCD *b* and SCD $b^{\prime }\in C_{i}$10:            $g_{n}=v\left(C_{i}\cup \left\{b\right\}\setminus \left\{b^{\prime }\right\}\right)+v\left(C_{j}\cup \left\{b^{\prime }\right\}\setminus \left\{b\right\}\right)-v\left(C_{i}\right)-v\left(C_{j}\right)$11:         **else if** $U_{C_{i}}+U_{b}\leq N$12:            $C^{\left(tmp\right)}\leftarrow$ SCD *b* joins $C_{i}$13:            $g_{n}=v\left(C_{i}\cup \left\{b\right\}\right)+v\left(C_{j}\setminus \left\{b\right\}\right)-v\left(C_{i}\right)-v\left(C_{j}\right)$14:         **if** $g_{n}>g$15:            $C^{\left(ok\right)}\leftarrow C^{\left(tmp\right)}$, $g=g_{n}$16:      $C^{\left(l\right)}\leftarrow C^{\left(ok\right)}$17:   l←l+118:**Output:** The set of clusters C

## 4. Subchannel Allocation (P2, P3) and Beamforming Design for MBSs (P4)

In this section, we propose methods to solve the SCA problems (6) and (7). Satisfactory subchannels are allocated to UEs with high channel gain while guaranteeing the constraints are met. In addition, the beamforming design for MBSs is also described.

### 4.1. Subchannel Allocation

(P2) For clusters, constraints (7b)–(7d) are the same as the constraints of an assignment problem where each task is assigned to only one worker to minimize the cost. One of the effective methods to solve assignment problems is the Hungarian method [31]. Compared with other methods, the Hungarian algorithm can solve large-scale assignment problems in polynomial time. Addtionally, it is a deterministic and global algorithm that guarantees the optimal solution is the best assignment. Regarding flexibility, the Hungarian method can be applied to solve assignment problems with equal or unequal numbers of resources and tasks, especially in the cases of variable numbers of network elements in clusters in our proposed SCA problem. Therefore, we use the Hungarian algorithm for making decisions in choosing the optimal subchannels for SUEs. Matrix ${\mathrm{VC}}_{maxprob}$, which consists of the value of ${\mathrm{VC}}_{u,n}$ with all pairs of SUE $u\in C_{i}$ and subchannel *n*, is given in Table 2.

To obtain the solution to the optimization problem (7), we use the Hungarian method in Algorithm 2 [30]. Because of the limitation of the number of UEs in each cluster by *N*, the complexity of Algorithm 2 is $O(N^{4})$ [31].
**Algorithm 2** The Hungarian method for SCA in each cluster.1:**Input**: VC matrix ${\mathrm{VC}}_{maxprob}$.2:Convert to minimization problem by subtracting the maximum of the matrix from all elements ${\mathrm{VC}}_{minprob}=\left\{{\mathrm{VC}}_{i,k}=\left|{\mathrm{VC}}_{i,k}-\max\left({\mathrm{VC}}_{maxprob}\right)\right||\;\;i=1,\ldots ,U_{C_{i}}\;\;\mathrm{and}\;\;k=1,\ldots ,N\right\}$.3:Extend ${\mathrm{VC}}_{minprob}$ to a square matrix with the size of N×N4:**Obtain modified matrix:**5:   Subtract the smallest entry from all entries in each row of ${\mathrm{VC}}_{minprob}$.6:   Subtract the smallest entry from all entries in each column of ${\mathrm{VC}}_{minprob}$.7:**Repeat**8:   Cover all zeros in the matrix with minimum number of vertical/horizontal lines $nl_{\min}$.9:   **if** $nl_{\min}<N$ **then**10:      Subtract the lowest number from all elements that are not covered by any line.11:      Add this lowest number to elements that are crossed by any two lines.12:**Until** $nl_{\min}=N$.13:Obtain the optimal association from zeros in the processed matrix.14:**Output**: The association matrix $X=\{x_{i,k}\;\;|\;\;i=1,\ldots ,,U_{C_{i}}\;\;\mathrm{and}\;\;k=1,\ldots ,N\}$.

(P3) For macrocells, constraint (6b) requires one MUE to be allocated only one subchannel. Moreover, the number of MUEs in a subchannel is not important due to the mMIMO MBSs. Therefore, the solution to the optimization problem (6) can be easily obtained by choosing the maximum value of ${\mathrm{VM}}_{m,n},n=1,\ldots ,N$ for each MUE *m*.

### 4.2. Beamforming Design

According to the model in Figure 2, after SCA, we need to design the beamforming vectors for MBSs. Assuming that the CSI is known at the MBSs, we introduce two types of appropriate beamforming vectors for two cases.

- If there are multiple MUEs that are served by MBS *f* in subchannel *n*, then the beamforming vector is one vector in the null space of the channel matrix from MBS *f* to all of its MUEs except MUE *m*, and is expressed as
  $$\begin{array}{c} w_{m,f}=\mathrm{Null}\left(H_{M_{f}\setminus m,f}^{\left(n\right)}\right). \end{array}$$
  Therefore, we can neglect the cross-talk interference in (2).
- If only one MUE *m* is served by MBS *f* in subchannel *n*, then according to the zero-forcing beamforming, the beamforming vector is as follows
  $$\begin{array}{c} w_{m,f}={\left(h_{m,f}^{\left(n\right)}\right)}^{†}/∥{\left(h_{m,f}^{\left(n\right)}\right)}^{†}∥. \end{array}$$
  In both cases, the magnitude of the beamforming vector is normalized to 1 ($\left|\right|w_{m,f}\left|\right|=1$). Thus, the PA for MBSs and SCDs only depends on $p_{m,f}^{\left(n\right)}$ and $p_{u,b}^{\left(n\right)}$.

## 5. Power Allocation for Macrocells and Small Cells (P5, P6)

Power allocation (PA) is a promising approach for interference mitigation in UDNs. In this section, we propose both a distributed method and a centralized method to obtain the solutions to the optimization problems (8) and (9). It is clear that the powers of MBSs and SCDs are variables in both optimization problems (8) and (9). This causes these optimization problems to be extremely complex and hard to solve. In addition, the channels from MBSs to MUEs are usually more strongly affected by environmental factors than the channel between SCDs and SUEs, while guaranteeing the QoS. Therefore, the optimization problem of MBSs has higher priority than that of SCDs. We assume that the interference from small-cell tiers to each MUE *m* is a constant at first as follows:

(12) $$I_{m,C}^{\left(n\right)}=\sum_{C_{i}\in C}\sum_{b\in C_{i}}\sum_{u\in U_{C_{i}}}a_{u,C_{i}}^{\left(n\right)}{\left|h_{m,b}^{\left(n\right)}\right|}^{2}P_{SCD}^{\max}/N.$$

Each active SCD uses the maximum power of $P_{SCD}^{\max}$, which is divided equally to th subchannels, to serve theSUEs. Thus, the interference from the small-cell tier to MUEs is extremely high when first solving the PA optimization problem of MBSs. This helps the QoS constraints to remain satisfied after obtaining the solution to the PA optimization problem of SCDs with very low interference.

### 5.1. Centralized Power Allocation

In this subsection, we propose practical methods to solve centralized PA problems for macrocells and small cells. In these methods, we combine three techniques: changing variables, approximating objective functions, and the Dinkelbach algorithm. To this end, the very complex maximizing EE optimization problems especially in UDNs are converted into much easier convex problems in iterative algorithms. This is a promising approach to achieving real-time computing in multitier massive-cell networks such as UDNs.

The EE maximization PA problem for macrocells is expressed as follows

(13a) $$\begin{array}{cc} \max_{p_{m,f}^{\left(n\right)}} & \;\frac{\sum_{m=1}^{M}\log_{2}\left(1+\frac{|h_{m,f}^{\left(n\right)}w_{m,f}{|}^{2}p_{m,f}^{\left(n\right)}}{I_{\mathrm{cross}-\mathrm{talk}}^{\left(n\right)}+I_{m,F\setminus f}^{\left(n\right)}+I_{m,C}^{\left(n\right)}+\sigma_{m}^{2}}\right)}{\sum_{f\in F}P_{f}^{total}}=\frac{\varphi_{M}\left(P_{F}\right)}{\pi_{M}\left(P_{F}\right)} \end{array}$$

(13b) $$\begin{array}{cc} s.t. & \;\sum_{n=1}^{N}\sum_{m\in M_{f}}a_{m,f}^{\left(n\right)}p_{m,f}^{\left(n\right)}\leq P_{\mathrm{MBS}}^{\max},\forall f\in F, \end{array}$$

(13c) $$\begin{array}{cc} \; & \;p_{m,f}^{\left(n\right)}\geq 0,\forall f\in F,\forall m\in M,n=1,\ldots ,N, \end{array}$$

(13d) $$\begin{array}{cc} \; & \;\gamma_{m}\geq \gamma_{m\;\min},\forall m\in M, \end{array}$$

where $\gamma_{m\;\min}≜2^{Tpm_{\min}/BW}-1$ is the minimum SINR to guarantee the QoS of MUEs. By using the beamforming vectors in Section 4.2, the value of cross-talk interference $I_{\mathrm{cross}-\mathrm{talk}}^{\left(n\right)}$ in the macrocells equals 0. $I_{m,C}^{\left(n\right)}$ is fixed by using estimation (12). We can easily convert constraint (13d) into a linear inequality becuase each $\gamma_{m}$ in (13d) is an affine-affine fraction. Therefore, all constraints in the optimization problem (13) are linear. Let $I_{m}\left(P_{F}\right)≜I_{\mathrm{cross}-\mathrm{talk}}^{\left(n\right)}+I_{m,F\setminus f}^{\left(n\right)}+I_{m,C}^{\left(n\right)}.$ Definitely, in (13a), a given UE *m* is served by MBS *f* in subchannel *n*, so $p_{m,f}^{\left(n\right)}>0$ with constraint (13d). For $x_{m}={\left|h_{m,f}^{\left(n\right)}w_{m,f}\right|}^{2}p_{m,f}^{\left(n\right)}$, $y_{m}=I_{m}\left(P_{F}\right)+\sigma_{m}^{2}$, and at the κ-th iteration ${\bar{x}}_{m}={\left|h_{m,f}^{\left(n\right)}w_{m,f}\right|}^{2}p_{m,f}^{\left(n)(\kappa \right)}$, ${\bar{y}}_{m}=I_{m}\left(P_{F}^{\left(\kappa \right)}\right)+\sigma_{m}^{2}$, where $P_{F}^{\left(\kappa \right)}$, is a feasible point of optimization problem (13). Following the method in [32] and using inequality (A1) in Appendix A.1, we have $\varphi_{M}\left(P_{F}\right)\geq {\tilde{\varphi }}_{M}\left(P_{F},P_{F}^{\left(\kappa \right)}\right),$ where ${\tilde{\varphi }}_{M}\left(P_{F},P_{F}^{\left(\kappa \right)}\right)≜\sum_{m=1}^{M}\left({\tilde{a}}_{m}^{\left(\kappa \right)}-{\tilde{b}}_{m}^{\left(\kappa \right)}/x_{m}-{\tilde{c}}_{m}^{\left(\kappa \right)}y_{m}\right)$ and ${\tilde{a}}_{m}^{\left(\kappa \right)}=\log_{2}\left(1+\frac{{\bar{x}}_{m}}{{\bar{y}}_{m}}\right)+\frac{2{\bar{x}}_{m}}{\ln\left(2\right)\left({\bar{x}}_{m}+{\bar{y}}_{m}\right)},$ ${\tilde{b}}_{m}^{\left(\kappa \right)}=$ $\frac{{\bar{x}}_{m}^{2}}{\ln\left(2\right)\left({\bar{x}}_{m}+{\bar{y}}_{m}\right)},\;{\tilde{c}}_{m}^{\left(\kappa \right)}=\frac{{\bar{x}}_{m}}{\ln\left(2\right){\bar{y}}_{m}\left({\bar{x}}_{m}+{\bar{y}}_{m}\right)}.$

Let $\lambda_{M}^{\left(\kappa \right)}≜\varphi_{M}\left(P_{F}^{\left(\kappa \right)}\right)/\pi_{M}\left(P_{F}^{\left(\kappa \right)}\right)$. According to the Dinkelbach algorithm, the suboptimization problem to find $P_{F}^{\left(\kappa +1\right)}$ for iteration (κ+1) is as follows

(14) $$\begin{array}{cc} \max_{p_{m,f}^{\left(n\right)}} & \;{\tilde{\varphi }}_{M}\left(P_{F},P_{F}^{\left(\kappa \right)}\right)-\lambda_{M}^{\left(\kappa \right)}\pi_{M}\left(P_{F}\right)\;s.t.\;\left(13b\right),\left(13c\right),\left(13d\right). \end{array}$$

Because ${\tilde{a}}_{m}^{\left(\kappa \right)},{\tilde{b}}_{m}^{\left(\kappa \right)},{\tilde{c}}_{m}^{\left(\kappa \right)}>0$, the objective function in (14), which is the sum of pairs of convex functions, i.e., constant×variable and −constant/variable (constant>0), is convex. Therefore, problem (14) is convex due to the convexity of the objective function and the affine functions at the constraints. The proof of the convergence is provided in Appendix B.1. Based on [33], the computational complexity of each iteration for solving (14) is $O\left({\left(MFN\right)}^{2}{\left(F+MFN+M\right)}^{2.5}+{\left(F+MFN+M\right)}^{3.5}\right)$ with the number of decision variables MFN and the number of constraints F+MFN+M.

It is given that the UE *u* is served by cluster $C_{i}$, which combines SCDs *b*. The EE maximization PA problem for small cells is given by

(15a) $$\begin{array}{cc} \max_{p_{u,b}^{\left(n\right)}} & \;\frac{\sum_{u=1}^{U}\log_{2}\left(1+\frac{\sum_{b\in C_{i}}|h_{u,b}^{\left(n\right)}{|}^{2}p_{u,b}^{\left(n\right)}}{I_{u,F}^{\left(n\right)}+I_{u,C\setminus C_{i}}^{\left(n\right)}+\sigma_{u}^{2}}\right)}{\sum_{C_{i}\in C}\sum_{b\in C_{i}}P_{b}^{\mathrm{total}}}=\frac{\varphi_{U}\left(P_{C}\right)}{\pi_{U}\left(P_{C}\right)} \end{array}$$

(15b) $$\begin{array}{cc} s.t. & \;\sum_{n=1}^{N}\sum_{u\in U_{C_{i}}}a_{u,C_{i}}^{\left(n\right)}p_{u,b}^{\left(n\right)}\leq P_{\mathrm{SCD}}^{\max},\forall b\in B, \end{array}$$

(15c) $$\begin{array}{cc} \; & \;p_{u,b}^{\left(n\right)}\geq 0,\forall b\in B,\forall u\in U,n=1,\ldots ,N, \end{array}$$

(15d) $$\begin{array}{cc} \; & \;\gamma_{u}\geq \gamma_{s\;\min},\forall u\in U, \end{array}$$

where $\gamma_{s\;\min}≜2^{Tps_{\min}/BW}-1$ is the minimum SINR that guarantees the QoS of the SUEs. Using the same technique as for the PA problem for macrocells, (15d) can be easily converted into a linear constraint. Therefore, the optimization problem (15) is a linear-constrained optimization problem. However, the objective function of the optimization problem (15) is more challenging than the optimization problem (13) of macrocells because there is more than one variable in the numerator of $\gamma_{u}$. Let $I_{u}\left(P_{C}\right)=I_{u,F}^{\left(n\right)}+I_{u,C\setminus C_{i}}^{\left(n\right)}.$ To find the lower bound of $\varphi_{U}\left(P_{C}\right)$, we use inequality (A3) in Appendix A.2. In detail, for $x_{u,b}={\left|h_{u,b}^{\left(n\right)}\right|}^{2}p_{u,b}^{\left(n\right)}$, $y_{u}=I_{u}\left(P_{C}\right)+\sigma_{m}^{2}$, and at the κth iteration ${\bar{x}}_{u,b}={\left|h_{u,b}^{\left(n\right)}\right|}^{2}p_{u,b}^{\left(n)(\kappa \right)}$, ${\bar{y}}_{u}=I_{u}\left(P_{C}^{\left(\kappa \right)}\right)+\sigma_{u}^{2}$, where $P_{C}^{\left(\kappa \right)}$ is a feasible point of the optimization problem (15). Following inequality (A3), we have $\varphi_{U}\left(P_{C}\right)\geq {\tilde{\varphi }}_{U}\left(P_{C},P_{C}^{\left(\kappa \right)}\right),$ where ${\tilde{\varphi }}_{U}\left(P_{C},P_{C}^{\left(\kappa \right)}\right)≜\sum_{u=1}^{U}\left({\tilde{a}}_{u}^{\left(\kappa \right)}-\sum_{b\in C_{i}}{\tilde{b}}_{u,b}^{\left(\kappa \right)}/x_{u,b}-{\tilde{c}}_{u}^{\left(\kappa \right)}y_{u}\right)$ and ${\bar{s}}_{u}=\sum_{b\in C_{i}}{\bar{x}}_{u,b},$ ${\tilde{a}}_{u}^{\left(\kappa \right)}=\log_{2}\left(1+\frac{{\bar{s}}_{u}}{{\bar{y}}_{u}}\right)+\frac{2{\bar{s}}_{u}}{\ln\left(2\right)\left({\bar{s}}_{u}+{\bar{y}}_{u}\right)},$ ${\tilde{b}}_{u,b}^{\left(\kappa \right)}=\frac{{\bar{x}}_{u,b}^{2}}{\ln\left(2\right)\left({\bar{s}}_{u}+{\bar{y}}_{u}\right)},\;{\tilde{c}}_{u}^{\left(\kappa \right)}=\frac{{\bar{s}}_{u}}{\ln\left(2\right){\bar{y}}_{u}\left({\bar{s}}_{u}+{\bar{y}}_{u}\right)}.$

Let $\lambda_{U}^{\left(\kappa \right)}≜\varphi_{U}\left(P_{C}^{\left(\kappa \right)}\right)/\pi_{U}\left(P_{C}^{\left(\kappa \right)}\right)$. According to the Dinkelbach algorithm, the suboptimization problem to find $P_{C}^{\left(\kappa +1\right)}$ for iteration (κ+1) is as follows

(16) $$\begin{array}{cc} \max_{p_{u,b}^{\left(n\right)}} & \;{\tilde{\varphi }}_{U}\left(P_{C},P_{C}^{\left(\kappa \right)}\right)-\lambda_{U}^{\left(\kappa \right)}\pi_{U}\left(P_{C}\right)\;s.t.\;\left(15b\right),\left(15c\right),\left(15d\right). \end{array}$$

**Figure 3:**
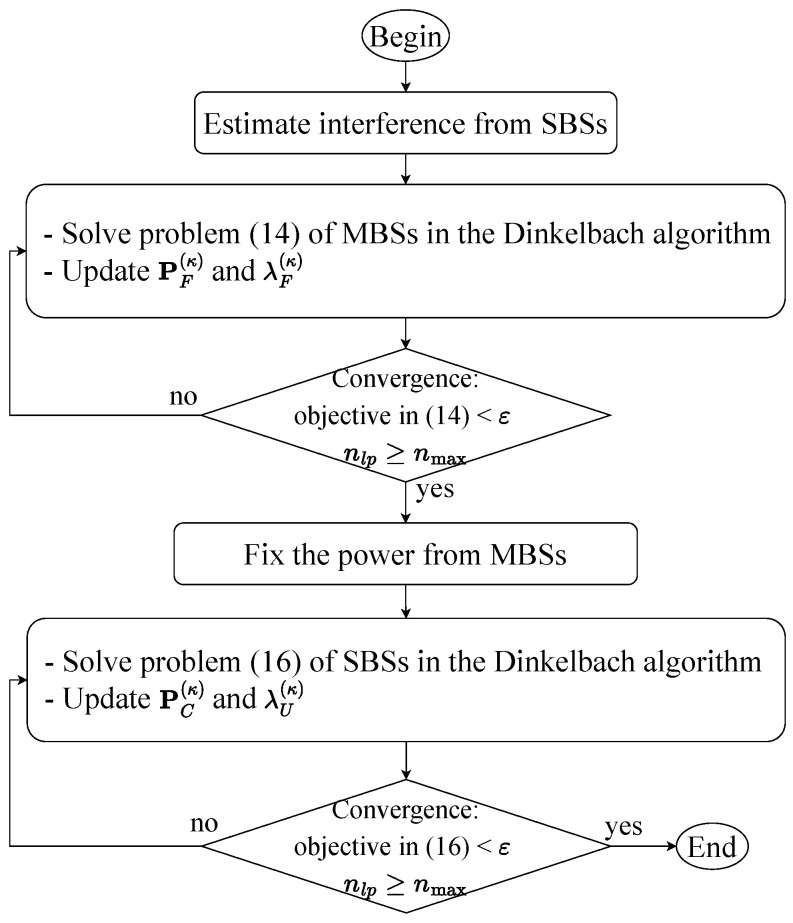
The flow chart of the proposed method to solve centralized PA.

The same as for optimization problem (14),  optimization problem (16) is also a convex problem. The proof of convergence is presented in Appendix B.2. Figure 3 shows a diagram for solving the centralized PA optimization problems, with ϵ denoting the tolerance value and $n_{\max}$ being the maximum number of iterations. If the values of the objective functions in both (14) and (16) are lower than the tolerance value ϵ or $n_{lp}\geq n_{\max}$, with $n_{lp}$ denoting the number of iterations, then the convergence holds true. With the number of decision variables BUN and the number of constraints B+BUN+U, the computational complexity of each iteration for solving (14) is $O\left({\left(BUN\right)}^{2}{\left(B+BUN+U\right)}^{2.5}+{\left(B+BUN+U\right)}^{3.5}\right)$.

### 5.2. Distributed Power Allocation by the Stackelberg Game

The solution of centralized PA methods is the best option for PA to MBSs and SCDs. However, when the number of small cells increases, the execution time of these methods also significantly rises. Therefore, centralized PA methods cannot satisfy the strict time constraints of UDNs in B5G. In this subsection, we propose a distributed PA method based on a SG to considerably reduce the complexity of the original optimization problems while retaining an acceptable efficiency of the solution. A SG is a type of NCG where players have no incentive to collaborate. There are two levels of priority in a SG. In detail, players with a high level of priority are called leaders and the others are followers. When starting a SG, leaders choose optimal actions first. Then, followers see the actions of the leaders to choose their optimal actions. Thus, a SG is also called a two-stage NCG.

The distance from MBSs to MUEs is usually much larger than the distance between SCDs to SUEs. Therefore, the problems of reflection, diffraction, and scattering influence the received signal at MUEs more than at SUEs. Meanwhile, MBSs must guarantee serving all UEs that are out of the coverage of the small cells, with the constraint of data rate. Therefore, MBSs, as leaders, have a high level of priority, and SCDs are followers in this SG.

#### 5.2.1. NCG for Leaders (MBSs)

We formulate the optimization problem (8) as an NCG, where $NCG_{leader}=\langle {\mathrm{PL}}_{F},P_{F},ul_{F}\rangle$, where ${\mathrm{PL}}_{F}=F$ is the set of players (i.e., MBSs), $P_{F}$ is a matrix of PA for all MBSs, and $ul_{F}\left(f\right)$ is the utility function of the EE of MUEs served by MBS *f*. The solution to $NCG_{leader}$ is a Nash equilibrium where no MBS has an incentive to change its power, and the power of each MBS is the best response to the power of other MBSs [30]. By using an NCG, the optimization problem (8) can be divided into multiple suboptimization problems for MBSs to obtain the best response for each player. In each subOP, we find an optimal solution for an MBS while the power of the other MBSs is fixed. A subOP for MBS *f* is formulated as

(17a) $$\begin{array}{cc} \max_{P_{f}} & \;ul_{F}\left(f\right)=\frac{\sum_{m\in M_{f}}\log_{2}\left(1+\gamma_{m}\right)}{P_{f}^{total}}=\frac{A_{f}\left(P_{f}\right)}{B_{f}\left(P_{f}\right)} \end{array}$$

(17b) $$\begin{array}{cc} s.t. & \;\sum_{n=1}^{N}\sum_{m\in M_{f}}a_{m,f}^{\left(n\right)}p_{m,f}^{\left(n\right)}\leq P_{\mathrm{MBS}}^{\max}, \end{array}$$

(17c) $$\begin{array}{cc} \; & \;p_{m,f}^{\left(n\right)}\geq 0,\forall m\in M_{f},n=1,\ldots ,N, \end{array}$$

(17d) $$\begin{array}{cc} \; & \;\gamma_{m}\geq \gamma_{m\;\min},\forall m\in M_{f}. \end{array}$$

The objective function (17a) is a concave–convex fraction. We can use the Dinkelbach method to solve the optimization problem (17). In detail, we introduce a new variable $\lambda_{f}$, and the optimization problem (17) can be rewritten as

(18) $$\begin{array}{cc} \max_{P_{f}} & \;UT_{f}\left(P_{f},\lambda_{f}\right)=A_{f}\left(P_{f}\right)-\lambda_{f}B_{f}\left(P_{f}\right)\;s.t.\;\left(17b\right),\left(17c\right),\left(17d\right). \end{array}$$

The objective function in (18) is a concave function in the maximization problem, so the optimization problem (18) is a convex problem. Therefore, the optimization problem (18) can be effectively solved by using CVX or CVXPY [34,35]. The optimal solution $P_{f}^{*}$ is also the optimal solution to optimization problem (18) if $UT_{f}\left(P_{f}^{*},\lambda_{f}^{*}\right)=0$. We use Algorithm 3 to find the solution for the NCG $NCG_{leader}$. With the number of decision variables $M_{f}N$ and the number of constraints $1+M_{f}N+M_{f}$, the computational complexity of each iteration for solving (18) is $O\left({\left(M_{f}N\right)}^{2}{\left(1+M_{f}N+M_{f}\right)}^{2.5}+{\left(1+M_{f}N+M_{f}\right)}^{3.5}\right)$. It is clear that the complexity of (18) is much lower than that of (14) because $M_{f}<M$ and only one MBS is considered.
**Algorithm 3** SG-based PA for macrocells and small cells.1:Initialize power matrix $P_{MBS}^{\left(0\right)}$ that meets constraints (17b), (17c), (17d), k=02:**while** the convergence is not reached3:   $P_{MBS}^{\left(k\right)}=P_{MBS}^{\left(k-1\right)}$4:   **for** f∈F5:      $P_{f}^{\left(tmp\right)}=P_{f}^{\left(k\right)}$6:      **while** $UT_{f}\left(P_{f}^{\left(tmp\right)},\lambda_{f}\right)>\epsilon$7:         $\lambda_{f}=A_{f}\left(P_{f}^{\left(tmp\right)}\right)/B_{f}\left(P_{f}^{\left(tmp\right)}\right)$, $P_{f}^{\left(tmp\right)}=P_{f}^{*}$ where $P_{f}^{*}$ is the solution of (18)8:      $P_{f}^{\left(out\right)}=P_{f}^{\left(tmp\right)}$9:   $P_{MBS}^{\left(k\right)}=P_{MBS}^{\left(out\right)}$, k=k+110:Initialize power matrix of SCDs $P_{cluster}^{\left(0\right)}$ that meets constraints (19b), (19c), (19d), k=011:**while** the convergence is not reached12:   $P_{cluster}^{\left(k\right)}=P_{cluster}^{\left(k-1\right)}$13:   **for** $C_{i}\in C$14:      $P_{C_{i}}^{\left(tmp\right)}=P_{C_{i}}^{\left(k\right)}$15:      **while** $UT_{C_{i}}\left(P_{C_{i}}^{\left(tmp\right)},\lambda_{C_{i}}\right)>\epsilon$16:         $\lambda_{C_{i}}=A\left(P_{C_{i}}^{\left(tmp\right)}\right)/B\left(P_{C_{i}}^{\left(tmp\right)}\right)$, $P_{C_{i}}^{\left(tmp\right)}=P_{C_{i}}^{*}$ where $P_{C_{i}}^{*}$ is the solution of (20)17:      $P_{C_{i}}^{\left(out\right)}=P_{C_{i}}^{\left(tmp\right)}$18:   $P_{cluster}^{\left(k\right)}=P_{cluster}^{\left(out\right)}$, k=k+1

#### 5.2.2. NCG for Followers (Clusters)

We formulate optimization problem (9) as an NCG $NCG_{follower}=\langle {\mathrm{PL}}_{C},P_{C},ul_{C}\rangle$, where ${\mathrm{PL}}_{C}=C$ is the set of players (i.e., clusters), $P_{C}$ is a matrix of PA for all clusters of SCDs, and $ul_{C}\left(C_{i}\right)$ is the utility function of the EE of cluster $C_{i}$. The PA problem for SCDs in (9) can be divided into multiple suboptimization problems by using NCG with players as clusters. A suboptimization problem of cluster $C_{i}$ is as follows

(19a) $$\begin{array}{cc} \max_{P_{C_{i}}} & \;ul_{C}\left(C_{i}\right)=\frac{\sum_{u\in U_{C_{i}}}\log_{2}\left(1+\gamma_{u}\right)}{\sum_{b\in C_{i}}P_{b}^{\mathrm{total}}}=\frac{A_{Ci}\left(P_{C_{i}}\right)}{B_{Ci}\left(P_{C_{i}}\right)} \end{array}$$

(19b) $$\begin{array}{cc} s.t. & \;\sum_{n=1}^{N}\sum_{u\in U_{C_{i}}}p_{u,b}^{\left(n\right)}\leq P_{\mathrm{SCD}}^{\max},\forall b\in C_{i}, \end{array}$$

(19c) $$\begin{array}{cc} \; & \;p_{u,b}^{\left(n\right)}\geq 0,\forall b\in C_{i},n=1,\ldots ,N, \end{array}$$

(19d) $$\begin{array}{cc} \; & \;\gamma_{u}\geq \gamma_{s\;\min},\forall u\in U_{C_{i}}. \end{array}$$

To obtain the best response of cluster $C_{i}$, the power of the other clusters is fixed. This best response is the solution to optimization problem (19). We use the Dinkelbach method to solve the optimization problem (19) because its objective function is a concave–convex fraction. In detail, we introduce a new variable $\lambda_{C_{i}}$, and the optimization problem (19) can be rewritten as

(20) $$\begin{array}{cc} \max_{P_{C_{i}}} & \;UT_{C_{i}}\left(P_{C_{i}},\lambda_{C_{i}}\right)=A_{Ci}\left(P_{C_{i}}\right)-\lambda_{C_{i}}B_{Ci}\left(P_{C_{i}}\right)\;s.t.\;\left(19b\right),\left(19c\right),\left(19d\right). \end{array}$$

The objective function in (20) is a concave function, so the optimization problem (20) is a convex optimization problem. Therefore, the optimization problem (20) can be effectively solved by using CVX or CVXPY [34,35]. The optimal solution $P_{C_{i}}^{*}$ is also the optimal solution to the optimization problem (20) if $UT_{C_{i}}\left(P_{C_{i}}^{*},\lambda_{C_{i}}^{*}\right)=0$. We use Algorithm 3 to find the solution to the NCG $NCG_{follower}$. With the number of decision variables $U_{C_{i}}C_{i}N$ and the number of constraints $C_{i}+U_{C_{i}}C_{i}N+U_{C_{i}}$, the computational complexity of each iteration for solving (20) is $O\left({\left(U_{C_{i}}C_{i}N\right)}^{2}{\left(C_{i}+U_{C_{i}}C_{i}N+U_{C_{i}}\right)}^{2.5}+{\left(C_{i}+U_{C_{i}}C_{i}N+U_{C_{i}}\right)}^{3.5}\right)$. The complexity of (20) is much lower than that of (16) since $U_{C_{i}}<U$ and $C_{i}<B$.

## 6. Simulation Results

Next, we investigated the performance of the proposed methods in a two-tier UDN. Three hexagon macrocells with a coverage radius of 100 m were deployed next to each other. In each macrocell, one MBS equipped with 128 antennas was located in the center of the cell. In addition, many small cells that had a radius of 20 m were randomly distributed by PPP. A small cell had an SCD equipped with one omnidirectional antenna. The UEs in the coverage of any small cell were served by the cluster that contained that small cell, and the others were served by theMBSs. The parameters in the simulations are given in Table 3 [36]. Additionally, we introduced two more clustering methods for comparison. In the traditional clustering method, each SUE is served by the nearest SCD. Meanwhile, with random clustering, each SUE randomly connected to an SCD, which had a distance to this SUE of less than d=3× the radius of small cells. On the other hand, equal power allocation, where the power of $P_{MBS}^{\max}/2$ of each MBS is equally divided among its MUEs and the power $P_{SCD}^{\max}$ of each active SCD is equally divided among the subchannels, was also used for comparison with the EE maximization PA methods. To prove the performance improvement, the proposed methods were compared with conventional methods based on multiple aspects such as convergence speed of PA methods, energy efficiency, sum rate, and transmit power usage. The acronyms for the combinations of clustering and PA methods are given in Table 4. To solve the optimization problems and build simulating programs, CVXPY and PYTHON were used [35]. The computing platform was a PC with CPU@3.7 GHz and 32 GB RAM memory.

**Figure 4:**
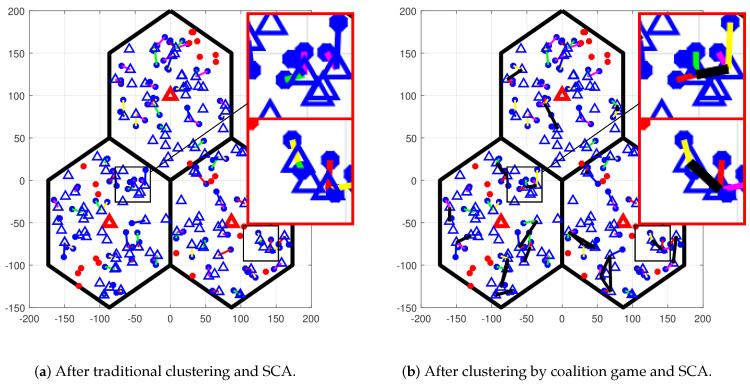
A typical triple-macrocell UDN after SCA with traditional and game-based clustering. Red triangles denote MBSs; red dots are MUEs; blue triangles are SCDs; blue dots are SUEs; SCDs in the same cluster are connected by black straight lines; SUEs served in the same subchannel are connected with their primal SCDs by the same color straight lines.

Figure 4 illustrates an example of a triple-macrocell UDN that combines 100 SUEs and 24 MUEs with a density of SCDs of 2000 SCDs/km${}^{2}$. In the first close-up in Figure 4a, two adjacent small cells use two same subchannels to serve their SUEs. This causes high interference for these SUEs. Meanwhile, after using the coalition game, these two small cells form a cluster to cooperatively transmit (the first close-up in Figure 4b). Therefore, there is no interference in a cluster. Another advantage of clustering that is shown in the second close-up is to enhance the quality of the desired signal. The SUE (red) subchannel is served by two small cells tgat have almost the same distance to it instead of being served by a single cell. When the density of SCDs is very high, these situations occur many times. Thus, clustering SCDs based on coalition game, and cooperative transmission help to efficiently mitigate intercell interference and improve the power of the received signal.

### 6.1. Convergence Speed of PA Methods

**Figure 5:**
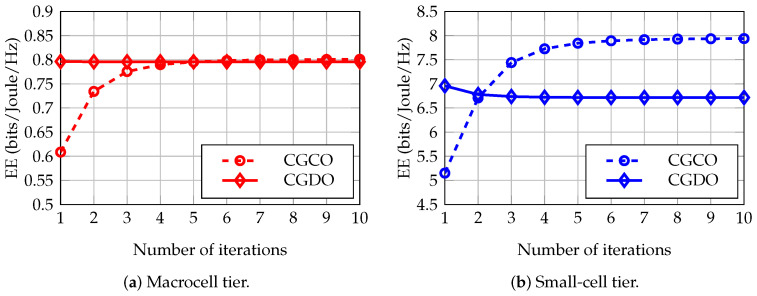
The convergence speed of the two PA methods.

In this subsection, we consider the convergence of CGCO and CGDO in the network that has an SCD density of 2000 SCDs/km${}^{2}$, 100 SUEs, 3 MBSs, and 24 MUEs. The changes and differences in the quantity of the EE of these methods, along with the iterations in the macrocell tier and small-cell tier, are shown in Figure 5a,b, respectively. It is given that one iteration of GT methods is complete after updating the best responses of all players. At the convergence, the EE of the CGDO is always lower than that of the CGCO. In detail, the EE of the CGDO is lower than that of CGCO in the macrocell tier and small-cell tier by 0.65% and 15.39%, respectively. However, the CGDO almost converges to the equilibrium after only one loop for updating all the best responses of the players, while it takes three and four iterations in the macrocell tier and small-cell tier, respectively, for the CGCO to achieve an EE of more than 95% at the convergence. On the other hand, in Figure 5b, the EE of the CGDO after one iteration is greater than the value of the convergence. This is reasonable because, in game theory, the value of the utility function at the Nash equilibrium may not be the best solution.

### 6.2. Performance Analysis of CGCO, CGDO, and TCDO

We evaluated the performance of the proposed methods regarding the EE, the sum rate, the total transmit power, and the execution time in different scenarios with the number of network elements that are given in Table 5, and the number of SUEs equaled 60% of the number of SCDs.

**Figure 6:**
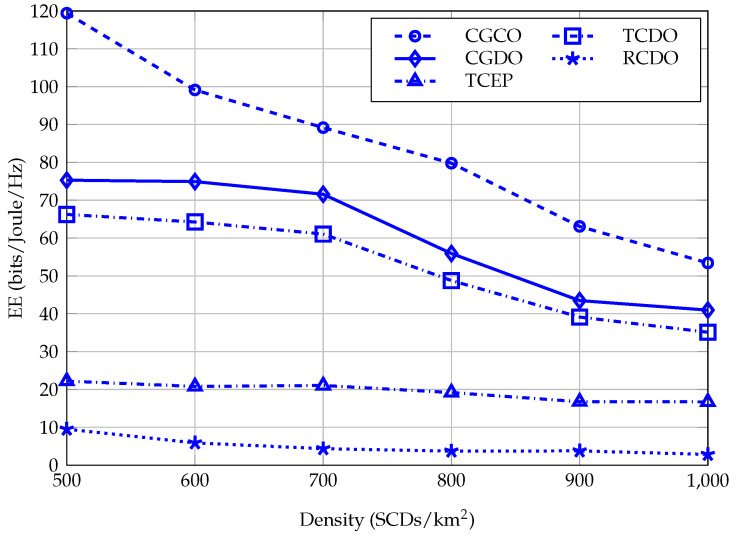
The changes in the EE of the methods in different scenarios.

Figure 6 displays data on the changes in the quantity of the EE of CGCO, CGDO, TCDO, RCDO, and TCEP in the small-cell tier when the density of SCDs increases from 500 to 1000 SCDs/km${}^{2}$. To overview, the EE of all methods witnesses a decrease with different levels since the denser the deployment of small cells is, the higher interference is. The EE of CGCO is greatest in both the five methods since it uses the centralized PA method. In detail, the EE of CGDO equals 74.09% of the EE of CGCO on average. Meanwhile, the EE of CGDO is greater from 11.15% to 17.23% than the one of TCDO in all testing scenarios. In addition, CGCO, CGDO, and TCDO outperform RCDO and TCEP in terms of the EE. Therefore, using the coalition game for clustering small cells before SCA and PA based on distributed method helps improve the efficiency of energy usage in UDNs compared with the traditional clustering and the random clustering.

Along with the EE, the computing execution time is an important factor to evaluate the performance of different methods. Table 6 describes the data in detail regarding the time required for the PA of CGCO, CGDO, and TCDO. Thanks to (12), the intensity of interference from small cells is pre-estimated in the PA optimization problems of the macrocell tier. Therefore, the execution time of the PA methods in the macrocell tier does not depend on the density of SCDs. On the other hand, the execution time for the PA of the MBSs of CGDO and TCDO is higher than the one of CGCO when the number of MUEs is small. The reason is that optimization problem (18) with the objective, a logarithmic function, takes a longer time to solve than optimization problem (16) with the objective as the sum of affine functions and rational functions when the number of variables is slightly different. However, the execution time in macrocells is less important than that in small cells with much more variables in the OPs. Considering the small-cell tier, when the density of the SCDs changes from 500 to 1000 SCDs/km${}^{2}$, the execution time of the CGCO significantly increases by 1.7 times, while those of the distributed methods CGDO and TCDO do not depend on the number of SCDs. Additionally, the execution time of CGCO is always longer, from 1.95 to 3.49 times that of CGDO. In large-scale UDNs, quick adaptation is key. If a very complex algorithm is used and the execution time is long, when the solution is obtained, it is not optimal anymore because the channel and user locations have changed too much. Therefore, the trade-off between EE and a quick execution time using our proposed PA method is essential and effective in UDNs.

**Figure 7:**
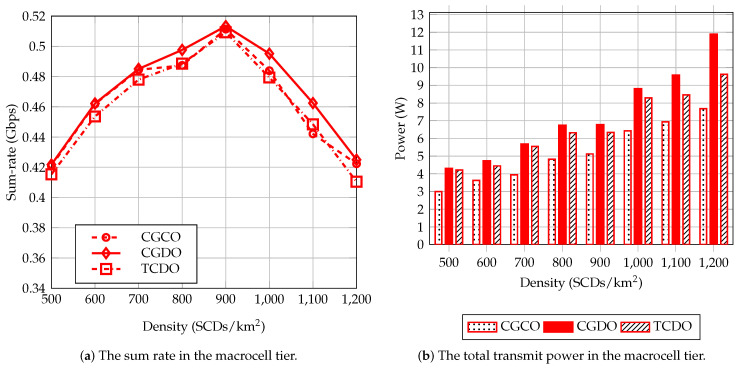
The sum rate and the total power consumption of the macrocells.

Figure 7 shows the average of the sum rate and the total power consumption of CGCO, CGDO, and TCDO in the macrocell tier of UDNs. The level of the sum-rate growth of the three methods gradually decreases when the number of small cells increases in a certain area. The sum rate of these methods increases and reaches the maximum at a density of 900 SCDs/km${}^{2}$. Then, it gradually decreases when densities are greater than 900 SCDs/km${}^{2}$ despite the increased power usage because MUEs experience extreme interference from the small-cell tier. To clearly identify this trend, we added two more scenarios with densities of 1100 and 1200 SCDs/km${}^{2}$, and we set the number of MUEs to 24. Additionally, in comparison with traditional clustering, SCDs were used in more subchannels for transmitting the signals to both their primal SUEs and the other SUEs in their clusters. This causes more interference from small cells to macrocells. Therefore, MBSs with CGDO use more power to achieve a higher sum rate than those of TCDO.

**Figure 8:**
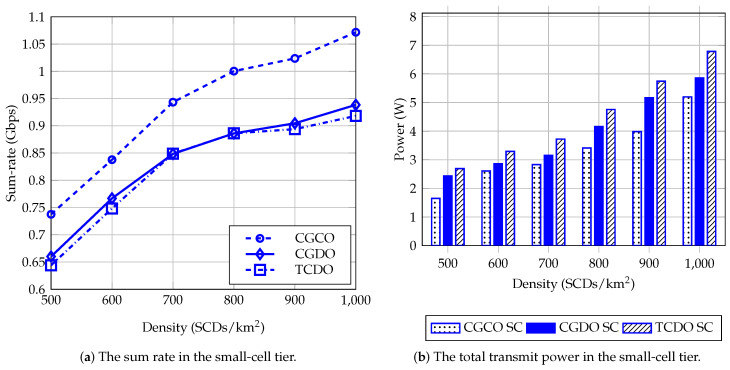
The sum rate and the total power consumption of the small cells.

Figure 8 describes the sum rate and the total transmit power of the three methods, namely CGCO, CGDO, and TCDO, in the small-cell tiers of UDNs with different network sizes. When the density of the SCDs increases, the energy consumption considerably increases, while the rate of increase in the sum rate declines. Due to using centralized optimization, the data rate of CGCO is always larger than those of the others, while the total transmit power of CGCO is the lowest for the three methods. The power consumption of CGDO is lower by 9.51% to 15.19% than that of TCDO. On the other hand, when the deployment of SCDs becomes denser, to achieve the same data rate, the SCDs use much more power. For example, in Figure 8a, the sum rates, which CGDO and TCDO achieve with small cells with densities of 800 and 900 SCDs/km${}^{2}$, are nearly equal. Meanwhile, the power consumption of CGDO and TCDO significantly increases in this range of density, as shown in Figure 8b. Moreover, the data rate of CGDO is slightly higher than that of TCDO. To summarize, the clustering method based on the coalition game helps to achieve a higher data rate than the traditional clustering method with lower power consumption.

### 6.3. Execution Time of Coalition Game (Algorithm 1) and the Hungarian Method (Algorithm 2)

Using the same simulation parameters as in Section 6.2, we evaluate the execution time of Algorithms 1 and 2 in Table 7. It is clear that the execution times of the coalition game and the Hungarian method increase with the increasing density of SCDs for the different levels. In detail, from 500 to 1000 SCDs/km${}^{2}$, the execution time of the coalition game increases 3.8 times and that of the Hungarian method increases 3.1 times. The computational complexity of the Hungarian method is $O(N^{4})$ in the worst case for the SCA in each cluster. Thus, its computational time has a linear relationship with the number of clusters in the small-cell tier.

## 7. Conclusions

We investigated the problem of resource allocation for UDNs with two tiers consisting of multiple MBSs and massive randomly distributed drones in this study. We proposed a paradigm that combines three processes: clustering using the coalition game, SCA using the Hungarian method, and PA using the SG as a distributed optimization method. Our simulations proved that the execution time of the centralized method for PA is much higher, by 1.95 to 3.49 times, than that of our distributed method, while the network EE of the proposed method using clustering based on the coalition game is improved by 11.15% to 17.23% compared with that of the distributed one with traditional clustering. Therefore, the centralized method is more suitable for UDNs with a small number of network elements, while the distributed method can be efficiently employed in large-scale UDNs where many MBSs and drones communicate simultaneously. Moreover, real-time computing plays an important role in drone-aided UDNs due to the mobility of both base stations (i.e., drones) and users. The execution time of the coalition game and the Hungarian method showed that they can be applied to real-time systems in UDNs.

For future work, with a massive number of users, data traffic is extremely high in drone-aided networks. To avoid network data congestion, drones equipped with caching storage, which can prestore common data packets, are a promising solution. However, the optimization problem for caching is integer programming with a large number of decision variables. Therefore, low-complexity algorithms for solving caching problems with the objective of minimizing the risk of network congestion are essential to be considered as an extension of this method. Additionally, to guarantee the quality of the signal received by users, drone-to-user communications need to be line of sight. Thus, the trajectory design for multiple drones in order to adapt to the movements of users is also an open topic to be addressed in the future.

## Figures and Tables

**Table 1:** The description of the main mathematical notation.

Notation	Definition
F	{1,2,…,F}: The set of MBSs
B	{1,2,…,B}: The set of SCDs
C	{1,2,…,C}: The set of clusters of SCDs
$C_{i}$	$\left\{1,2,\ldots ,C_{i}\right\}$: The set of SCDs in cluster *i*
U	{1,2,…,U}: The set of SUEs
M	{1,2,…,M}: The set of MUEs
$U_{C_{i}}$	$\left\{1,2,\ldots ,U_{C_{i}}\right\}$: The set of SUEs served by cluster $C_{i}$
$U_{b}$	$\left\{1,2,\ldots ,U_{b}\right\}$: The set of SUEs served by SCD *b*
$M_{f}$	$\left\{1,2,\ldots ,M_{f}\right\}$: The set of MUEs served by MBS *f*
$s_{m,f}^{\left(n\right)}$	∈C: The instant desired symbol of MUE *m* served by MBS *f* in subchannel *n*
$p_{m,f}^{\left(n\right)}$	$E\{s_{m,f}^{\left(n\right)}s_{m,f}^{(n)H}\}$: The transmit power of MBS *f* to MUE *m* in subchannel *n*
$s_{u,b}^{\left(n\right)}$	∈C: The instant desired symbol of SUE *m* served by SCD *b* in subchannel *n*
$p_{u,b}^{\left(n\right)}$	$E\{s_{u,b}^{\left(n\right)}s_{u,b}^{(n)H}\}$: The transmit power of SCD *b* to SUE *u* in subchannel *n*
$h_{m,f}^{\left(n\right)}$	$\in C^{1\times T}$: The channel response from MBS *f* to MUE *m* in subchannel *n*
$h_{m,b}^{\left(n\right)}$	∈C: The channel response from interference source SCD *b* to MUE *m* in subchannel *n*
$h_{u,b}^{\left(n\right)}$	∈C: The channel response from SCD *b* to SUE *u* in subchannel *n*
$h_{u,f}^{\left(n\right)}$	$\in C^{1\times T}$: The channel response from interference source MBS *f* to SUE *u* in subchannel *n*
$w_{m,f}$	$\in C^{T\times 1}$: Beamforming vector at MBS *f* to serve MUE *m*
$a_{m,f}^{\left(n\right)}$, $a_{u,C_{i}}^{\left(n\right)}$	subchannel allocation indicator
$y_{m,f}^{\left(n\right)}$	Received signal at MUE *m* served by MBS *f* in subchannel *n*
$y_{u,C_{i}}^{\left(n\right)}$	Received signal of SUE *u* served by cluster $C_{i}$ in subchannel *n*
$I_{u,F}^{\left(n\right)}$	$\sum_{f\in F}\sum_{m\in M_{f}}a_{m,f}^{\left(n\right)}{\left|h_{u,f}^{\left(n\right)}w_{m,f}\right|}^{2}p_{m,f}^{\left(n\right)}$: The interference power from MBSs
$I_{u,C\setminus C_{i}}^{\left(n\right)}$	$\sum_{C_{j}\in C\setminus C_{i}}\sum_{b^{\prime }\in C_{j}}\sum_{u^{\prime }\in U_{C_{j}}}a_{u^{\prime },C_{j}}^{\left(n\right)}{\left|h_{u,b^{\prime }}^{\left(n\right)}\right|}^{2}p_{u^{\prime },b^{\prime }}^{\left(n\right)}$: The interference power from other clusters except cluster $C_{i}$
$I_{\mathrm{cross}-\mathrm{talk}}^{\left(n\right)}$	$\sum_{m^{\prime }\in M_{f}\setminus m}a_{m^{\prime },f}^{\left(n\right)}{\left|h_{m,f}^{\left(n\right)}w_{m^{\prime },f}\right|}^{2}p_{m^{\prime },f}^{\left(n\right)}$: The cross-talk interference power from MBS *f*
$I_{m,F\setminus f}^{\left(n\right)}$	$\sum_{f^{\prime }\in F\setminus f}\sum_{m^{\prime }\in M_{f^{\prime }}}a_{m^{\prime },f^{\prime }}^{\left(n\right)}{\left|h_{m,f^{\prime }}^{\left(n\right)}w_{m^{\prime },f^{\prime }}\right|}^{2}p_{m^{\prime },f^{\prime }}^{\left(n\right)}$: The interference power from MBSs except MBS *f*
$I_{m,C}^{\left(n\right)}$	$\sum_{C_{i}\in C}\sum_{b\in C_{i}}\sum_{u\in U_{C_{i}}}a_{u,C_{i}}^{\left(n\right)}{\left|h_{m,b}^{\left(n\right)}\right|}^{2}p_{u,b}^{\left(n\right)}$: The interference power from the clusters of SCDs
$\mathrm{CN}(0,\sigma^{2})$	A complex Gaussian random variable with zero mean and variance $\sigma^{2}$

**Table 2:** The matrix of values of ${\mathrm{VC}}_{u,n}$.

SUE∖Channel	1	2	…	N
1	${\mathrm{VC}}_{1,1}$	${\mathrm{VC}}_{1,2}$	…	${\mathrm{VC}}_{1,N}$
2	${\mathrm{VC}}_{2,1}$	${\mathrm{VC}}_{2,2}$	…	${\mathrm{VC}}_{2,N}$
…	…	…	…	…
$U_{C_{i}}$	${\mathrm{VC}}_{U_{C_{i}},1}$	${\mathrm{VC}}_{U_{C_{i}},2}$	…	${\mathrm{VC}}_{U_{C_{i}},N}$

**Table 3:** Simulation parameters.

Parameter	Numerical Value
Carrier frequency/Total bandwidth	2 GHz/20 MHz
Bandwidth per subchannel	4 MHz
# subchannels	5
Path loss from MBSs to UEs	$128.1+37.6\log_{10}d$ [dB], *d* in km
Path loss from SCDs to UEs	$140.7+36.7\log_{10}d$ [dB], *d* in km
Shadowing standard deviation	8 dB
Noise power density	−174 dBm/Hz
Maximum transmit power of MBS	46 dBm
Maximum transmit power of SCD	30 dBm
Circuit power of MBS	$20\%P_{MBS}^{\max}$
Circuit power of SCD	$20\%P_{SCD}^{\max}$
The radius of a macrocell	100 m
The radius of a small cell	20 m
# transmit antennas per MBS	128
The minimum throughput per MUE	0.1 Mbps
The minimum throughput per SUE	1 Mbps

**Table 4:** ID of strategies for comparison.

ID	Clustering	PA for Macrocells	PA for Small Cells
CGCO	Coalition Game	Centralized optimization	Centralized optimization
CGDO	Coalition Game	Game-based optimization	Game-based optimization
TCDO	Traditional clustering	Game-based optimization	Game-based optimization
RCDO	Randomly clustering	Game-based optimization	Game-based optimization
TCEP	Traditional clustering	Equal power allocation	Equal power allocation

**Table 5:** The different network size scenarios.

Density (SCDs/km${}^{2}$)	500	600	700	800	900	1000
# MBSs	3	3	3	3	3	3
# MUEs	9	12	15	18	21	24

**Table 6:** The average execution time for solving one optimization problem of PA methods for different network sizes.

Density (SCDs/km${}^{2}$)	500	600	700	800	900	1000
CGCO MC (ms)	113	129	148	163	183	195
CGCO SC (ms)	243	284	323	364	379	411
CGDO MC (ms)	157	165	168	176	183	180
CGDO SC (ms)	125	124	124	122	115	118
TCDO MC (ms)	160	176	171	176	180	180
TCDO SC (ms)	118	112	114	109	103	104

**Table 7:** The execution time of the clustering-method-based coalition game and SCA-based Hungarian method for different network sizes.

Density (SCDs/km${}^{2}$)	500	600	700	800	900	1000
Coalition game (μs)	282.2	309.1	426.7	543.4	586.9	1073.8
The Hungarian method (μs)	542.5	734.5	816.4	925.8	976.6	1688.6

## Data Availability

The data presented in this study are available on request from the corresponding author.

## Appendix A. Fundamental Inequalities

### Appendix A.1

According to [32,37], we have

(A1) $$\begin{array}{ccc} \log_{2}\left(1+x/y\right) & \geq & \tilde{a}-\tilde{b}/x-\tilde{c}y, \end{array}$$

where $\tilde{a}=\log_{2}\left(1+\bar{x}/\bar{y}\right)+2\bar{x}/\left(\ln\left(2\right)\left(\bar{x}+\bar{y}\right)\right)>0,$ $\tilde{b}={\bar{x}}^{2}/\left(\ln\left(2\right)\left(\bar{x}+\bar{y}\right)\right)>0,$ $\tilde{c}=\bar{x}/\left(\ln\left(2\right)\left(\bar{x}+\bar{y}\right)\bar{y}\right)>0.$

### Appendix A.2

Functions $h:R^{n}\to R$ and $g:R^{n}\to R^{n}$, $h\left(u\right)≜\log_{2}\left(\sum_{i=1}^{n}\exp\left(u_{i}\right)\right)\;\mathrm{and}\;g_{i}\left(x_{i}\right)≜\ln\left(1/x_{i}\right)$ are both convex in $x={\left[x_{i}\right]}_{i=1}^{n}>0$ [38]. The extended-value extension $\tilde{h}$ of *h* is a nondecreasing function in each argument. Therefore, the vector composition of functions *g* and *h* is as follows

$$\begin{array}{cc} f(x) & ≜h\left(g(x)\right)=h\left(g_{1}\left(x_{1}\right),\ldots ,g_{n}\left(x_{n}\right)\right)=\log_{2}\left(\sum_{i=1}^{n}\exp\left(g_{i}\left(x_{i}\right)\right)\right)=\log_{2}\left(\sum_{i=1}^{n}1/x_{i}\right), \end{array}$$

is convex. In addition, if we add a convex function to f(x), then the convexity is preserved, and the new convex function is expressed as

$$\begin{array}{cc} f_{1}\left(x,y\right) & ≜f\left(x\right)+\log_{2}\left(\frac{1}{y}\right)=\log_{2}\left(\sum_{i=1}^{n}\frac{1}{x_{i}}\right)+\log_{2}\left(\frac{1}{y}\right)=\log_{2}\left(\sum_{i=1}^{n}\frac{1}{x_{i}y}\right). \end{array}$$

On the other hand, if $z_{0}$ is a positive real number, we have an inequality as follows [39]

(A2) $$\log_{2}\left(1+z\right)\geq \alpha \log_{2}\left(z\right)+\beta ,$$

where $\alpha =z_{0}/\left(1+z_{0}\right),\beta =\log_{2}\left(1+z_{0}\right)-\alpha \log_{2}\left(z_{0}\right).$ The left part of (A2) is close to another part, and the equality holds true at $z=z_{0}$ [39].

Using (A2) and the convexity of $f_{1}\left(x,y\right)$, we have

$$\begin{array}{cc} f_{2}\left(x,y\right)≜ & \log_{2}\left(1+\sum_{i=1}^{n}\frac{1}{x_{i}y}\right)\geq \bar{\alpha }\log_{2}\left(\sum_{i=1}^{n}\frac{1}{x_{i}y}\right)+\bar{\beta }\\ \geq & \bar{\alpha }\left(f_{1}\left(\bar{x},\bar{y}\right)+\langle \nabla f_{1}\left(\bar{x},\bar{y}\right),\left(x,y\right)-\left(\bar{x},\bar{y}\right)\rangle \right)+\bar{\beta }\\ = & \underset{\tilde{a}}{\underbrace{\bar{\alpha }f_{1}\left(\bar{x},\bar{y}\right)+\bar{\beta }+\bar{\alpha }\sum_{i=1}^{n}\frac{1}{\ln\left(2\right)\bar{y}\bar{x_{i}}\sum_{i=1}^{n}\left(1/\left(\bar{x_{i}}\bar{y}\right)\right)}+\bar{\alpha }\frac{\sum_{i=1}^{n}\left(1/\bar{x_{i}}\right)}{\ln\left(2\right)\bar{y}\sum_{i=1}^{n}\left(1/\left(\bar{x_{i}}\bar{y}\right)\right)}}}\\ \; & -\sum_{i=1}^{n}\underset{\tilde{b_{i}}}{\underbrace{\frac{1}{\ln\left(2\right)\bar{y}{\bar{x_{i}}}^{2}\sum_{i=1}^{n}\left(1/\left(\bar{x_{i}}\bar{y}\right)\right)}}}x_{i}-\underset{\tilde{c}}{\underbrace{\frac{\sum_{i=1}^{n}\left(1/\bar{x_{i}}\right)}{\ln\left(2\right){\bar{y}}^{2}\sum_{i=1}^{n}\left(1/\left(\bar{x_{i}}\bar{y}\right)\right)}}}y, \end{array}$$

where $\bar{\alpha }=\left(\sum_{i=1}^{n}\frac{1}{\bar{x_{i}}\bar{y}}\right)/\left(1+\sum_{i=1}^{n}\frac{1}{\bar{x_{i}}\bar{y}}\right),\bar{\beta }=\log_{2}\left(1+\sum_{i=1}^{n}\frac{1}{\bar{x_{i}}\bar{y}}\right)-\bar{\alpha }\log_{2}\left(\sum_{i=1}^{n}\frac{1}{\bar{x_{i}}\bar{y}}\right).$ Replacing $x_{i}\to 1/x_{i}$ and ${\bar{x}}_{i}\to 1/{\bar{x}}_{i}$ in $f_{2}\left(x,y\right)$, we have

(A3) $$\begin{array}{c} \log_{2}\left(1+\sum_{i=1}^{n}x_{i}/y\right)\geq \tilde{a}-\sum_{i=1}^{n}\tilde{b_{i}}/x_{i}-\tilde{c}y. \end{array}$$

where $\tilde{a}=\log_{2}\left(1+\frac{\bar{s}}{\bar{y}}\right)+\frac{2\bar{s}}{\ln\left(2\right)\left(\bar{s}+\bar{y}\right)},{\tilde{b}}_{i}=\frac{{\bar{x}}_{i}^{2}}{\ln\left(2\right)\left(\bar{s}+\bar{y}\right)},$ $\tilde{c}=\frac{\bar{s}}{\ln\left(2\right)\bar{y}\left(\bar{s}+\bar{y}\right)},\bar{s}=\sum_{i=1}^{n}{\bar{x}}_{i}.$

## Appendix B. Proving the Convergence

### Appendix B.1

Let $P_{F}^{\left(\kappa +1\right)}$ be the solution of the convex optimization problem (14). Thus, the value of objective function in (14) at $P_{F}^{\left(\kappa +1\right)}$ is greater than the value of the other feasible points (i.e., $P_{F}^{\left(\kappa \right)}$) as [32]

(A4) $$\begin{array}{c} {\tilde{\varphi }}_{M}\left(P_{F}^{\left(\kappa +1\right)},P_{F}^{\left(\kappa \right)}\right)-\lambda_{M}^{\left(\kappa \right)}\pi_{M}\left(P_{F}^{\left(\kappa +1\right)}\right)>{\tilde{\varphi }}_{M}\left(P_{F}^{\left(\kappa \right)},P_{F}^{\left(\kappa \right)}\right)-\lambda_{M}^{\left(\kappa \right)}\pi_{M}\left(P_{F}^{\left(\kappa \right)}\right). \end{array}$$

From (A1), the equality is achieved when $x=\bar{x}$ and $y=\bar{y}$. Therefore, we have

(A5) $$\begin{array}{c} {\tilde{\varphi }}_{M}\left(P_{F}^{\left(\kappa \right)},P_{F}^{\left(\kappa \right)}\right)-\lambda_{M}^{\left(\kappa \right)}\pi_{M}\left(P_{F}^{\left(\kappa \right)}\right)=\varphi_{M}\left(P_{F}^{\left(\kappa \right)}\right)-\lambda_{M}^{\left(\kappa \right)}\pi_{M}\left(P_{F}^{\left(\kappa \right)}\right)=0. \end{array}$$

With (A4) and (A5), we have

(A6) $$\begin{array}{c} \lambda_{M}^{\left(\kappa +1\right)}=\frac{\varphi_{M}\left(P_{F}^{\left(\kappa +1\right)}\right)}{\pi_{M}\left(P_{F}^{\left(\kappa +1\right)}\right)}\geq \frac{{\tilde{\varphi }}_{M}\left(P_{F}^{\left(\kappa +1\right)},P_{F}^{\left(\kappa \right)}\right)}{\pi_{M}\left(P_{F}^{\left(\kappa +1\right)}\right)}>\lambda_{M}^{\left(\kappa \right)}. \end{array}$$

Therefore, the first loop in Figure 3 converges at least to a local maximal point of the optimization problem (13).

### Appendix B.2

Using the same approach as in Appendix II.1, we have $\lambda_{U}^{\left(\kappa +1\right)}>\lambda_{U}^{\left(\kappa \right)}.$ From this, the second loop in Figure 3 also at least converges to a local maximal point of the optimization problem (15).
